# Editorial: Emerging Biomarkers in Genitourinary Tumors

**DOI:** 10.3389/fonc.2019.00326

**Published:** 2019-04-26

**Authors:** Rodolfo Montironi, Matteo Santoni, Alessia Cimadamore, Antonio Lopez-Beltran, Liang Cheng

**Affiliations:** ^1^Section of Pathological Anatomy, School of Medicine, United Hospitals, Polytechnic University of the Marche Region, Ancona, Italy; ^2^Oncology Unit, Macerata Hospital, Macerata, Italy; ^3^Department of Pathology and Surgery, Faculty of Medicine, Cordoba University, Cordoba, Spain; ^4^Department of Pathology and Laboratory Medicine, Indiana University School of Medicine, Indianapolis, IN, United States

**Keywords:** genitourinary tumors, microbiome, renal cell carcinoma, prostate cancer, bladder cancer, liquid biopsy, PSMA, immunotherapy

This is a contemporary update in the field of *Emerging Biomarkers in Genitourinary Tumors*. This series of papers, published in Frontiers in Oncology, section Genitourinary Oncology, by internationally renowned researchers, covers five major topics: (1). Identification of immunological biomarkers in genitourinary cancers; (2). New prostate cancer targets for imaging and therapy; (3) Liquid molecular biomarkers in genitourinary tumors; (4) Emerging biomarkers in testicular germ cell tumors; and (5) Future perspectives on molecular biomarkers in genitourinary tumors: toward a personalized approach to diagnosis, prognosis and prediction of response to therapy.

The first two papers are related to the Identification of immunological biomarkers in genitourinary cancers. In particular, the first, by Cimadamore et al., deals with the biological relationship between the gut microbiome and the immune system, in particular cancer development and treatment. As an example, Akkermansia muciniphila is a commensal associated with excellent clinical outcomes in renal cell carcinoma and non-small cell lung cancer. Interesting results have emerged on the microbiome in prostate cancer (PCa) patients, with specific bacteria as potential biomarkers in risk stratification. Abnormal gut microbiome composition could also have an influence on primary resistance to PD-1 blockade in mice xenografts and patients with cancer. The contribution by Lopez-Beltran et al. deals with the identification of novel immunological biomarkers in kidney cancers. Robust and reliable biomarkers are crucial for patient's selection for treatment with immunomodulatory drugs. PD-L1 expression is predictive of better response from both PD-1 and PD-L1 inhibitors in a variety of tumor types including RCC. A single biomarker for patient selection may not be feasible, given that immune responses are dynamic and evolve over time. A multidisciplinary approach is very much needed to fully develop the current and future value of immune checkpoint inhibitors in clinical practice.

The third paper of the whole series, by Cimadamore et al., deals with New PCa targets for imaging and therapy, focusing on Prostate-Specific Membrane Antigen (PSMA). This contribution reviews the current role of PSMA as a marker for PCa diagnosis, imaging and therapy. PSMA is expressed in the epithelial cells of the prostate and is strongly upregulated in PCa, with elevated expression correlating with androgen independence, metastasis and progression. PSMA has been found to be an active target of investigation by several approaches, including the successful use of small molecule inhibitors, RNA aptamer conjugates, PSMA-based immunotherapy, and PSMA-targeted prodrug therapy. The next three papers deals with Liquid molecular biomarkers in genitourinary tumors. The first of the three, by Di Nunno et al., is related to recent advances in liquid biopsy in patients with castration resistant PCa. The selection of patients more likely to benefit from a specific therapeutic approach still remains a key issue as well as the early identification of patients with aggressive disease which could benefit from a more aggressive treatment strategy. They review the literature to explore current knowledge on liquid biopsy in PCa focusing on possible future applications. In particular they focus on circulating DNA and circulating tumor cells as a promising and attractive approach despite to date practical applications of these techniques are few and not validated. The paper by Santoni et al. is an updates on urine markers in superficial and non-superficial bladder cancer (BCa). There is a growing evidence toward the use of minimally invasive “liquid biopsy” to identify new biomarkers. DNA- and RNA-based markers in body fluids such as blood and urine are promising potential markers in diagnostic, prognostic, predictive and monitoring BCa. However, proteomic and genomic data must to be validated in well-designed multicenter clinical studies, before to be employed in clinic oncology. The paper by Santoni et al. deals with recent findings and future challenges of circulating tumor cells (CTCs) in renal cell carcinoma (Santoni et al.). Renal Cell Carcinoma (RCC) may absolutely benefit from the development of non-invasive and reliable biomarkers, allowing early and timely personalized treatment changes. The introduction of CTC analysis within daily clinical practice for patients with RCC seems still so far at the moment. However, the advances obtained in the last 5 years in isolating and analyzing CTCs bring optimism about the future therapeutic landscape in RCC patients.

The contribution by Chovanec et al. deals with Emerging biomarkers in testicular germ cell tumors (GCTs). The ability to predict prognosis and treatment response in GCTs did not improve for many years. Clinical trials with novel targeting agents that were conducted in refractory GCT patients have proven to have negative outcomes. Novel biomarkers have emerged in the field of GCT oncology. Since then, oncology has exploded with various molecular biomarkers to further refine the prognosis and treatment of malignancies. This review summarizes the current knowledge in the research of novel biomarkers in GCTs.

The remaining two papers deal with Future perspectives on molecular biomarkers in genitourinary tumors: toward a personalized approach to diagnosis, prognosis and prediction of response to therapy. The paper by Giulietti et al. is related to emerging biomarkers in BCa identified by network analysis of transcriptomic data. Such complex gene interaction networks can be revealed by a recently developed systems approach called Weighted Gene Co-expression Network Analysis (WGCNA). In this review, the authors focused on the studies where the WGCNA approach has been applied to analyze gene expression data deriving from BCa samples. The paper by Giunchi et al. is a perspective article on emerging molecular technologies in genitourinary tumors. In particular, they deal with wide spectrum mutational analyses using next generation sequencing (NGS) platforms that will soon represent the standard-of-care technologies for the assessment of genetic variants in genitourinary tumors. They also deal with genome-wide trascriptome analyses which include gene expression profiling, miRNA and non-coding RNA profiling and RNA sequencing. Toward the end of the contribution they refer to patient-derived xenografts (PDX), i.e., mouse models where disaggregated cells or little fragments of human tumors are implanted into immunodeficient mice. The establishment of a PDX allows treating and monitoring the response to treatment of the original tumor *in vivo* in the mouse, instead of the patient, providing the best therapeutic selection at the same time.

## Conclusions

The identification of effective biomarkers has becoming a major focus in cancer research, mainly due to the necessity of selecting potentially responsive patients in order to improve their outcomes, as well as to reduce the toxicity and costs related to ineffective treatments. This Research Topic aims to include the description of these emerging techniques and identify the most promising biomarkers in genitourinary tumors.

## Author Contributions

RM and MS: Conception and design. AC: Drafting the manuscript. AL-B and LC: Critical revision of the manuscript and review of the literature.

### Conflict of Interest Statement

The authors declare that the research was conducted in the absence of any commercial or financial relationships that could be construed as a potential conflict of interest.

